# Cultural competence of dutch physician assistants: an observational cohort study

**DOI:** 10.1186/s12909-023-04112-8

**Published:** 2023-03-03

**Authors:** Susanne Leij-Halfwerk, Daniëlla van Uden, Sophie J. A. Jooren, Geert van den Brink

**Affiliations:** 1 Master Physician Assistant, HAN University of Applied Sciences, Verlengde Groenestraat 75, 6525EJ Nijmegen, The Netherlands; 2 Diakonessenhuis Hospital, Herculesplein 32-34, 3584 AA Utrecht, P.O. Box 80250, 3508 TG Utrecht, The Netherlands

**Keywords:** Cultural competence, Physician assistant, Healthcare, Education

## Abstract

**Background:**

Current cultural competence training needs were assessed as baseline measurement in Dutch physician assistant (PA) students and PA alumni that were not specifically trained in cultural competence. In particular, differences in cultural competency between PA students and PA alumni were assessed.

**Methods:**

In this cross-sectional, observational cohort study knowledge, attitude, and skills and self-perceived overall cultural competence were assessed in Dutch PA students and alumni. Demographics, education and learning needs were collected. Total cultural competence domain scores as well as percentage of maximum scores were calculated.

**Results:**

A total of 40 PA students and 96 alumni (female:75%; Dutch origin:97%) consented to participate. Cultural competence behavior was moderate in both groups. In contrast, general knowledge and exploration of patients’ social context were insufficient, i.e., 53% and 34%, respectively. Self-perceived cultural competence was significantly higher in PA alumni (6.5 ± 1.3, mean ± SD) than in students (6.0 ± 1.3; *P* < 0.05). Low heterogeneity among PA students and educator exists. Seventy percent of the respondents considers cultural competence important and the majority expressed a need for cultural competence training.

**Conclusions:**

Dutch PA students and alumni have moderate overall cultural competence, but insufficient knowledge and exploring social context. Based on these outcomes the curriculum of the master of science program for physician assistant will be adapted.Emphasis should be made to increase the diversity of PA students to stimulate cross-cultural learning and developing a diverse PA workforce.

## Background

Patient ethnicity predicts the quality of care one receives, independent of access to health care or socioeconomic status [1]. With the increasing migrant populations in the Netherlands – from 20% in 2009 to 24% in 2020 [2] – medical healthcare needs to be adapted to the requirements of this culturally and/or ethnically diverse patient population [3]. Barriers for equity in healthcare lay within poorly matched care to the minority needs, language, cultural familiarity, or health systems [1]. Health professionals and professional organizations subscribe to this view and should take a leadership role in advocating for interventions to reduce these disparities, although it is not yet clear yet what works best [4, 5].

Doctor-patient communication is directly linked to patient satisfaction, adherence to treatment and, subsequently, health outcomes [6]. In a context where health professionals increasingly engage diverse patients with different perspectives of health the patient communication is challenging and requires culturally competent health professionals [7]. Cultural competency has been defined as “the ability of healthcare professionals to communicate with and effectively provide high-quality care to patients from diverse sociocultural backgrounds” [7] is essential to professionalism and quality of care [8]. Culturally competent health professionals improved public health and patient satisfaction and may have a positive effect on the outcome of medical treatment [9].

Training of cultural competency for health professionals has been suggested to improve their cultural behavior [10] and increasing awareness of provider bias and discrimination in medical decision-making has been observed [7, 11]. Cultural competence has been gradually incorporated in the Physician Assistant (PA) programs since about two decades in the US [12] and since 2017 in the Netherlands [13]. Cultural competency education for the PA is similar to that used in medical education and mainly focuses on knowledge, attitudes and skills [14].

Although cultural competence and cross-cultural training in PA programs showed increased multicultural awareness, knowledge and skills [15, 16], study results were heterogeneous as various instruments or constructs of cultural competence had been used. Nevertheless, it was uniformly stated that exposure to diversity and cultural issues is essential to develop cultural desire and awareness. Recently, data in US PA students showed that they acknowledge the importance of cultural competency in their profession, but also acknowledge their own lack of knowledge and skills on this topic [17]. Cultural competence of Dutch PAs is not known, although data on Dutch medical students and physicians identified gaps in knowledge and culturally competent behavior [18]. Therefore, it was suggested that cultural competence training and creating awareness of students’ incompetence should be part of the medical training program [18].

With regard to the urgent need of cultural competence training of PA students, the curriculum of the master physician assistant will be adapted and monitored for efficacy. To determine educational needs, current cultural competence was assessed as baseline measurement in Dutch PA students and PA alumni that were not specifically trained in cultural competence. In particular, differences in cultural competence between PA students and PA alumni were determined.

## Methods

### Study design

This was a cross-sectional, observational study to quantitatively assess baseline cultural competence in a cohort of PA students and alumni of the master of science program at the HAN University of Applied Sciences, Nijmegen, The Netherlands.

#### Participants

PA students and alumni who were not formal trained on cultural competence during their PA training were recruited in August 2020 among students from cohort 2019 and 2020 (*n* = 149) and the alumni database of the HAN University of Applied Sciences (*n* = 412). Participants were invited by e-mail to participate in the study.

Participation was voluntary and without any restrictions. Responses were collected anonymously, and no personal information was to be collected. This study was deemed exempt from scientific medical research involving human subjects according to the Dutch law (‘WMO’) and medical ethical approval for this study was therefore not obligatory. The study protocol was reviewed by the Ethical Research Committee of the HAN University of Applied Science (Reference: ECO 189.06/20) for local approval. Consent of the participants was obtained online prior to the start of the cultural competence questionnaire.

#### General data of the cohort

Demographic data included gender, PA status, working experience, country of origin of participants and their parents, professional experience with minority patients, cultural competence courses and (current) working location.

#### Assessment of cultural competence

Cultural competence was assessed using a questionnaire based upon the conceptual framework for teaching and learning of cultural competence including knowledge, attitude, and skills [18]. Cultural competence was assessed by three domains: i) General knowledge of ethnic minority care provision and interpretation services, ii) Reflection ability (attitude) for insight into one’s own understanding of prejudice and cultural frames of reference determined by the Groningen Reflection Ability Scale (GRAS) [19], and iii) Cultural competent consultation behavior (skills) during medical consultations with ethnic minority patients.

The original items on knowledge were updated to the current state of the art and the short case scenarios were adapted to match with the PA profession. Finally, self-perceived overall cultural competence was assessed using a 1–10 scale.

#### Education and learning needs of the PA

PAs experiences during university education on cultural competency, data on cultural diversity of students and teachers, and experiences on the role of PA education in culturally competency were collected. Usefulness and learning needs on cultural competence in relation to relevant competencies of the PA curriculum were explored.

#### Data collection and storage

Data was collected using a web-based questionnaire developed in Qualtrics^©^ XM software (Qualtrics, Provo, Utah, USA; version August 2020). The data will be stored digitally for a maximum duration of 10 years at the HAN University on a password-protected research drive. Access to the research data is limited to researchers of the study.

### Statistical analyses

Response rates were determined by calculating the ratio of responders to the number of invited PA students and alumni. Cultural competence scores were summed per dimension for each domain. Relative scores were calculated as percentage of maximum scores for each dimension and interpreted as insufficient cultural competence when < 60%, moderate when 60–80%, and sufficient when > 80% [18]. Descriptive statistics were performed and presented where applicable. Chi-square tests were performed to analyse demographic characteristics between PA students and alumni. Differences between mean or median group scores per dimension were analyzed using either parametric or non-parametric statistical tests for differences in cultural competence between PA students and alumni. *P*-values < 0.05 were considered statistically significant.

## Results

### Demographics

From August to September 2020 a total of which 136 consented to complete the online questionnaire (response rate 27%), the majority being PA alumnus (*n* = 96), female (*n* = 98) and of Dutch origin (*n* = 132) (Table 1).

**Table 1:** Demographics of Dutch physician assistant (PA) students and alumni

**Variable**		**Total** *n* = 136	**PA students** *n* = 40	**PA alumni** *n* = 96	P*
Gender, n (%)	Female	98 (72)	30 (75)	68 (71)	0.62
	Male	38 (28)	10 (25)	28 (29)	
Born in the Netherlands, n (%)	Yes	132 (97)	40 (100)	92 (96)	0.19
	No	4 (3)	0 (0)	4 (4)	
Parents					
born in the Netherlands, n (%)	Mother	130 (96)	39 (98)	91 (95)	0.48
	Father	134 (99)	40 (100)	94 (98)	0.36
≥ 1 parent/caregiver completed university, n (%)	Yes	60 (44)	18 (45)	42 (44)	0.89
	No	76 (56)	22 (55)	54 (56)	
Health care working experience, n (%)	0–5 yr	9 (7)	8 (20)	1 (1)	0.00**
	6–10 yr	20 (15)	12 (30)	8 (8)	
	11–15 yr	19 (14)	6 (15)	13 (14)	
	15 + yr	88 (65)	14 (35)	74 (77)	
PA working experience, n (%)	0–5 yr	73 (54)	40 (100)	33 (34)	N.A
	6–10 yr	27 (20)	-	27 (28)	
	11–15 yr	25 (18)	-	25 (26)	
	15 + yr	11 (8)	-	11 (12)	
Minority patients in work area, n (%)	< 5%	14 (10)	4 (10)	10 (10)	0.44
	5–10%	35 (26)	6 (15)	29 (30)	
	11–25%	31 (23)	10 (25)	21 (22)	
	26–50%	18 (13)	5 (13)	13 (14)	
	> 50%	4 (3)	2 (5)	2 (2)	
	Unknown	34 (25)	13 (33)	21 (22)	

^*^PA students versus PA alumni, Chi-Square test; N.A.: not applicable

^**^One cell has < 5 observations

Fifteen percent of the responders had an experience of living abroad for half a year or more. The majority (65%) had a working experience in health care of 15 years or more being statistically significant higher in alumni and 54% had a PA working experience up to 5 years. Nineteen percent had previously worked in one of the largest cities within the Netherlands. The number of minority patients was estimated to be 25% or less in the working area of 60% of the respondents (Table 1).

### Cultural competence

The questions on cultural competence domains were completed by at least 80% of the respondents. Although moderate scores were obtained for both PA students and alumni, insufficient general knowledge on care provision of ethnic minorities was observed in both groups (Table 2).

**Table 2:** Cultural competence of Dutch physician assistant (PA) students and alumni

		**Total**	**PA students**	**PA alumni**	***P*** *
**Cultural competence domains**					
Knowledge		*n* = 115	*n* = 31	*n* = 84	
*General ethnic minority care provision*	Mean (SD) % of max. score^†^	4.3 (1.5) 53	4.3 (1.4) 54	4.2 (1.6) 53	0.86
*Interpretation services*	Mean (SD) % of max. score	3.2 (1.0) 63	3.0 (1.0) 61	3.2 (1.0) 64	0.43
Reflection Ability		*n* = 109	*n* = 29	*n* = 80	
*GRAS score*^‡^	Mean (SD) % of max. score	91 (7) 79	90 (5) 78	91 (7) 79	0.59
Culturally competent consultation behavior		*n* = 124	*n* = 35	*n* = 89	
*Exploring perspective*	Mean (SD) % of max score	2.7 (1.1) 68	2.7 (0.8) 67	2.7 (1.1) 68	0.68
*Exploring health literacy*	Mean (SD) % of max. score	3.1 (1.1) 79	3.0 (1.1) 76	3.2 (1.2) 80	0.06
*Exploring social context*	Mean (SD) % of max. score	1.0 (0.7) 34	0.8 (0.5) 27	1.1 (0.7) 37	0.42
*Use of interpretation services*	Mean (SD) % of max. score	4.9 (1.0)** 82	4.9 (1.1)** 82	4.9 (1.0)** 82	0.95
**Self-perceived cultural competence**^**ǂ**^		*n* = 136	*n* = 40	*n* = 96	
	Mean (SD)	6.3 (1.3)	6.0 (1.2)	6.5 (1.3)	0.038

^*^PA students versus PA alumni, Mann Whitney U test

^**^Total, *n* = 117; PA students, *n* = 31; PA alumni, *n* = 86

^†^Cultural competence as percentage of maximum score: < 60% insufficient, 60–80% moderate, and > 80% sufficient

^‡^Groningen Reflection Ability Scale (maximum score: 23–115)

^ǂ^Score 1–10

**Fig. 1:**
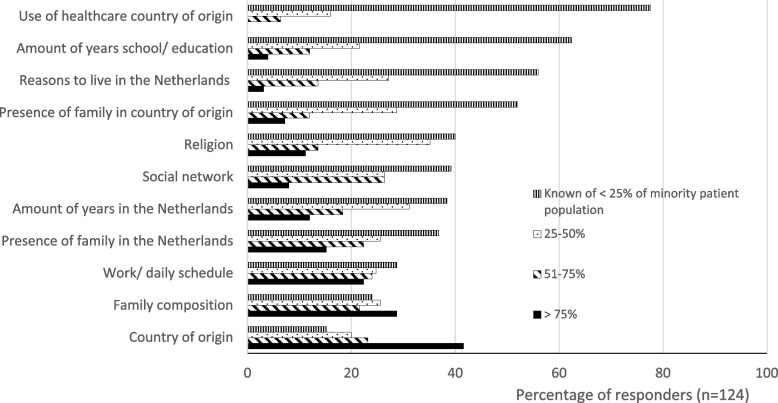
Cultural consultation behaviour of Dutch physician assistants – exploring social context of minority patients in the consultation practice

Reflection ability was moderate for all PAs. Also, moderate scores for consultation behavior were detected, although exploring of patients’ social context was insufficient (Table 2). In particular, only 20 to 40% of the PAs were aware of the family composition or country of origin of more than 75% of the minority patients in their practice (Fig. 1). Moreover, the majority of the PAs (56 to 78%) knows of less than 25% of the minority patients in their practice details about the use of health care in the migrants’ country of origin, the number of school years, or their reasons for migration. Finally, the self-perceived cultural competence of all PAs was rated as moderate, although alumni rated themselves significantly higher (6.5 ± 1.3, mean ± SD) compared to students (6.0 ± 1.2, P < 0.04) (Table 2).

### Language barriers and communication

**Fig. 2:**
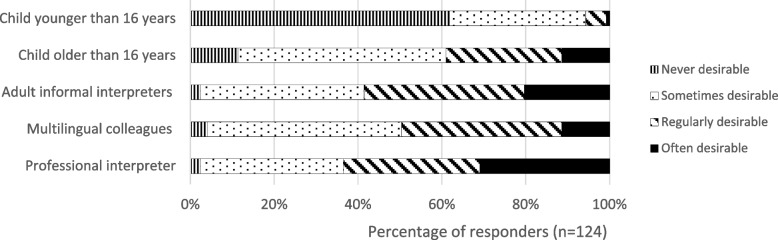
Desirability of using different types of interpreters according to Dutch physician assistants

Thirty-nine percent of all PAs often to regularly encountered language difficulties when consulting minority patients in the year before the study and 78% had experienced using a professional interpreter. The PAs had sufficient interpreter behavior during consultation (82% of maximum score, Table 1). Respondents’ desirability for using a professional interpreter is presented in Fig. 2. According to the majority of the PAs the use of a professional interpreter is regularly to often preferable, and 62% indicated the use of a child younger than 16 years as never desirable.

### Educational needs for cultural competence and the PA curriculum

A total of 111 respondents completed the questions on the PA curriculum and educational needs. According to twelve percent of all PAs the PA curriculum has added value to their cultural competence behavior. Only 29% of the PAs indicated to feel confident in health care consultation of minority patients, whereas only eight percent felt they had received sufficient cultural competence training during the PA education. Sixty percent indicated that having a different cultural background than their patients did not cause any problems during consultations. Nevertheless, 48% of the PAs acknowledged that there is a need for healthcare professionals with various cultural backgrounds within the Netherlands to provide the best possible care. Moreover, 70% of the PAs considered cultural competence important for their work as PA.

Sixty-seven and 78% of the PA student and alumni, respectively, indicated to have had few intercultural diversity among fellow students during their health care bachelor and PA master education. Even so, PA educators were little culturally heterogeneous according to 78% of the PA students and alumni. Ninety-two percent of those having experienced extracurricular cultural competence training indicated that this added to their culturally competent behaviour. Forty-three percent of all PA respondents indicated a need for training to increase their knowledge of culturally competent medical care and treatment of patients from diverse cultural backgrounds. The majority of the respondents indicated to have a moderate to high educational need regarding the competencies of the PA curriculum on medical treatment and social approach that are related to a culturally diverse patient population (Table 3).

**Table 3:**
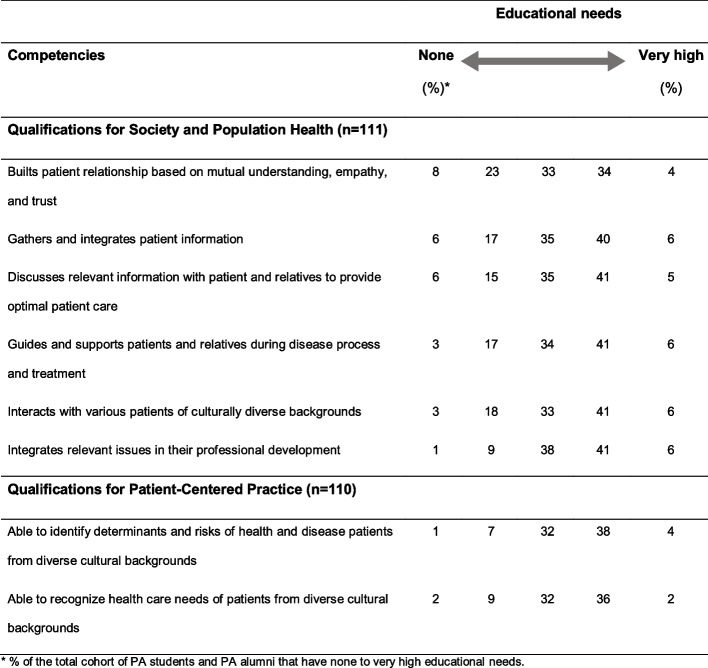
Educational needs of Dutch PA students and alumni for cultural competence related to core competences of the PA master curriculum [20]

## Discussion

This study showed that culturally competent behavior of a cohort of Dutch PA students and alumni from the HAN University of Applied Sciences was moderate, although general knowledge on ethnic minority care provision and exploring social context during consultation was insufficient. Only self-perceived cultural competence was higher in PA alumni compared to students. PAs considered cultural competence essential for providing quality of healthcare and expressed a need for education in this field. In particular, cultural heterogeneity among peer students and teachers was considered low.

To our best knowledge, this is the first study on cultural competency of PAs in the Netherlands. Cultural competency of Dutch PAs was comparable with those in the US as well as Dutch medical students and physicians showing cultural competence being considered important, but students being deficient in cultural knowledge, skills and behavior during encounters [17, 18]. The low scores on cultural knowledge of Dutch PA students and alumni may be explained by the fact that cultural competence has only recently been added to the PA program in the Netherlands [13]. The current study showed PAs’ self-perceived cultural competence to be moderate, with values being significantly higher in alumni, which could be explained by the higher number of working years in the alumni group. In-practice exposure to a culturally diverse population is essential for development of cultural awareness and culturally competent behavior [21].

Culturally competent consulting behavior was moderate in this sample of Dutch PA students and alumni and health literacy was moderately explored. However, exploration of patients’ social context was very low in both groups. Not knowing this information of patients is of concern as low literacy is associated with several adverse health outcomes [22]. In the model of shared-decision making in the intercultural context, professionals should develop skills to recognize possible differences in language, values about health and illness, expectations and prejudices [23]. So, although PAs value themselves for being moderate cultural competent, the gap in exploration of the social context of minority patients and recognizing communication limitations may limit patient-centered care [23]. Also, good patient-centered communication requires overcoming language difficulties and PAs should be taught that high-quality care to patients with language difficulties is possible when effort in using interpreters is made [24].

Cultural diversity in the PA curriculum should be taught with appropriate theoretical foundation and context, and show that cultural competence can be a vehicle for improving health care in general [25].The respondents in our study were culturally aware and indicated to be highly interested in cultural competence training, which has previously been reported for PAs [17]. Although physicians may value cross-cultural care as important, behavior in practice shows otherwise: little time to address cultural issues for training, formal evaluation or role modeling [26] or existence of subtle biases based on ethnicity [27]. So, the importance of educating physicians to treat patient with equity, not equally, is clear.

Intercultural communication should be part of the intercultural training as well and include language differences, differences in perception of illness and disease, social components of health communication, and doctors’ and patients’ prejudices and assumptions [28]. More specific, the three core communication skills, i.e. listening, exploring and checking, should be part of the medical curriculum [29]. Consulting with a professional interpreter should be practiced, as previously addressed, as well as teaching knowledge on mechanisms relevant to various ethnic groups [29]. Observed consultations of Dutch physicians with non-Dutch patients showed that physicians practice only generic communication skills or some relevant intercultural communication skills and focus mainly on the biomedical aspects [30]. In our study, reflections skills as measured by the GRAS [19] were rated high by all physician assistants and were similar to those in Dutch medical students and residents [18]. The scores reflect well-developed general reflection skills in these professionals, however, no insight in actual reflection on their own prejudices or cultural values is given [18]. In addition, there may be a difference between residents’ self-perception and their actual performance, as was previously observed [31]. Paternotte et.al. [32] suggested to train specific skills such as asking about the language proficiency of patients or checking if the proposed treatment plan fits into the cultural habits of the patient. This is in line with the results of our study, as exploring these elements of the social context of minority patients was often omitted.

The PA cohort in the present study was homogeneous, of Dutch origin and also had experienced very little cultural diversity during their education regarding peers and educators. Comparable figures have been previously reported in the US [33, 34] as well as in the Netherlands [35] and is a topic of concern [36]. For the improvement of cultural competence organizational and structural interventions are necessary in addition to the clinical education initiatives that have been discussed above [7]. Increasing diversity in the healthcare profession including the PA workforce will increase the possibility to eliminate health disparities [1] as it is (more) representing the general population [33, 37]. An ethnically and culturally diverse student population will improve cross-cultural learning and bring diversity of thought into the classroom [33, 38]. Educators should stimulate awareness of personal biases and an open attitude [29].

### Study limitations

The response-rate among PAs was quite low and the questionnaire was completed by 74% (*n* = 100) of the respondents. Particularly, respondents tended to drop-out during completing the GRAS questionnaire. Therefore, the results of the study may be biased representing mainly motivated PAs. To obtain a more representative and larger sample the data should preferably be collected concomitant with education hours. In addition, regression analysis would add significantly to the impact of this paper to control for various demographic variables, particularly with a larger sample, but was beyond the scope of the study. A challenge for future research is standardization of assessing cultural competence. Many different cultural competency assessment tools exist but non being validated in PAs or even in other professions, hampering good comparison between the outcomes of studies performed. As the construct of cultural competence in the other regions such as the US is different compared to the Netherlands, and used for different health professions with different meanings [39] it is difficult to comparing cultural competence of PAs between the Netherlands and the US. Nevertheless, the results of this study can be well compared with those in physicians studies in the Netherlands, as the questionnaire used in this study was originally developed for use in Dutch medical students [18] and slightly adapted for the purpose of this study.

Self-perceived cultural competence assessments, used in the majority of studies, are subject to reporting bias and not suitable for rigorous cultural competence measurement and education on a uniform cultural competency construct [12]. So more emphasis should be made on observation methods for cultural competent behavior [40] or objective structured clinical exam [16, 41]. In addition, a curriculum-scan such as the Tool for Assessing Cultural Competence Training (TACCT) could provide insight in the content of the curriculum [42] and provide suggestions for improvement. Finally, the term cultural competence is subject to discussion as it includes more than knowledge only. Knowledge is an essential, but not the exclusive, aspect for health care professionals to becoming aware of the patients’ culture and of one’s own, facilitating patient-centered care. Thus, future studies should focus on cultural humility as well.

## Conclusions

This study shows that cultural competence in a cohort of Dutch PA students and alumni was moderate but have insufficient general knowledge on ethnic minority care provision and exploring social context. PAs acknowledge the importance of cultural competence essential for providing quality of healthcare and expressed a need for education in this field. Based on these outcomes the curriculum of the master of science program for physician assistant will be adapted and monitored for efficacy. As cultural competence is variously defined, educated, and measured, uniformity between PA curricula should be encouraged and objective measures developed. Simultaneously, emphasis should be made to increase the diversity of PA students to stimulate cross-cultural learning as well as develop a diverse PA workforce.

## Data Availability

The datasets used and/or analysed during the current study are available from the corresponding author on reasonable request.

## Abbreviations

GRAS: Groningen reflection ability scale
PA: Physician assistant
TACCT: Tool for assessing cultural competence training
